# Usage patterns of blue flower color representation by Encyclopedia of Life content providers

**DOI:** 10.3897/BDJ.2.e1143

**Published:** 2014-08-11

**Authors:** Chantal-Marie Wright, Katja C Seltmann

**Affiliations:** † American Museum of Natural History, New York, United States of America

**Keywords:** Encyclopedia of Life, TraitBank, color models and analysis, user phenotype annotation, Phenotypic Quality Ontology

## Abstract

Encyclopedia of Life (EOL) is a resource for community-driven biodiversity data, focusing on species information and images. Research into blue flowers to compare color ('blueness') at different elevations revealed that data content providers describe flowers as blue for any color hue in the range from blue to magenta. We propose methods for standardizing color values and color searching within EOL by means of an expanded color vocabulary and improved access to image metadata, in order to improve the research capacity of this valuable resource.

## Introduction

The stated mission of Encyclopedia of Life (Parr et al. 2014b) is "To increase awareness and understanding of living nature through an Encyclopedia of Life that gathers, generates, and shares knowledge in an open, freely accessible and trusted digital resource" (http://EOL.org/about). In addition to data uploaded directly to EOL by Curators (http://eol.org/info/curators), content is aggregated from a large number of websites known as Content Partners (http://eol.org/content_partners). These partners include expert-driven scientific databases and museum collections, as well as collectively edited websites such as Wikipedia.

Several prior studies noted the prevalence of blue flowers in high elevation areas (Weevers 1952, Brunet 2009). These studies focus on specific geographic areas and are not worldwide or continental in scope. To address the question of whether blue flowers are an adaption prevalent at high elevations, aggregated data from worldwide resources is needed. This study annotated the 'blueness' of blue flowers at all elevations in North America from photographs available from the EOL. Only images of flowers known to be blue in color were sampled, as we were concerned with the amount of blue present in a flower at different elevations, and not the distribution of all flower colors. During the investigation it became apparent that searching for the color "blue" resulted in flower images with a range of color hues. We then compared the color representation of each retrieved image using three different color models including the Red, Green and Blue (RBG; Adobe Systems Inc. 2012); Hue, Saturation, and Value (HSV; Smith 1978); and Phenotypic Quality Ontology (PATO; Gkoutos et al. 2005) class representation.

## Material and methods

*Data Acquisition*

**Figure 1.**
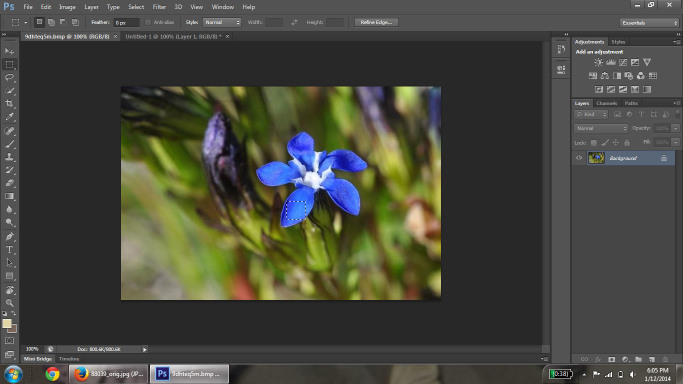
Representative area of petal selected (*Gentiana nivalis*).

**Figure 2.**
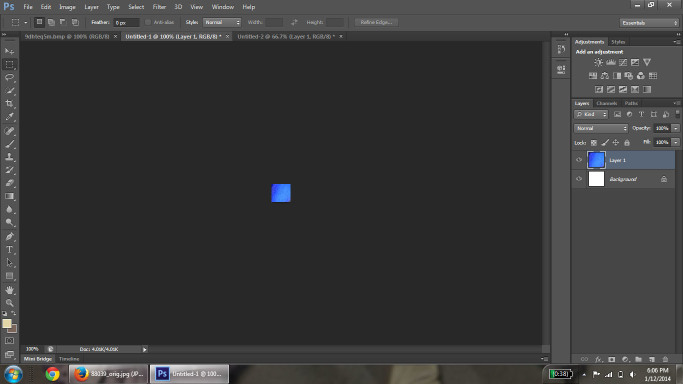
Selected area of petal copied and opened in new window.

**Figure 3.**
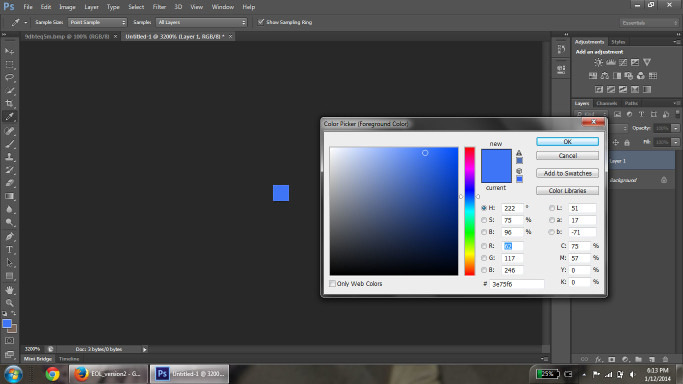
Selected area has been resized to 1 pixel; color picker tool shows the RGB values of the pixel.

Images used in the analysis were retrieved from the Encyclopedia of Life (EOL; Parr et al. 2014b). The image search was conducted through the EOL portal search interface with "no filter" selected to obtain the broadest search criteria possible. The search term entered each time was "blue flower." An initial list of North American species was determined from the image search, and non-plant results were discarded. The native range of the species was determined using the US Agricultural Department PLANTS Database (USDA, NRCS, National Plant Data Team 2014). After a complete list of EOL available native North American species was compiled, the images from each species were collected. We randomly sampled up to 10 images for each species from the entire set of retrieved images for that species. In addition, the first image from each locality was selected; if there were less than 10 localities, a second picture from each locality was acquired. Priority was given to non-cultivated localities over botanical and personal gardens. After selection, average RGB values were calculated for each image using Adobe Photoshop CS6 Extended program (Adobe Systems Inc. 2012). Photoshop was selected as the program of choice based on its wide availability and ease of use, as well as its ability to average color across pixels. To this end, the petal(s), or representative area, of the flower were selected and copied (Fig. 1) and opened in a new image window (Fig. 2). The selected petal areas were then condensed using the "Image Size" tool to a size of 1×1 pixel. This had the effect of 'averaging' the color of the petals. The "Eyedropper" tool was used on this single pixel to display the RGB values of the pixel with the color picker tool (Fig. 3) and the corresponding RGB values were recorded. If the flower had several petals that were different in color, a sample from each petal was selected, copied and pasted in a row on a blank new image; this row of samples was then selected and re-sized for averaging.

*Data Analysis*

Data analysis and graphing were performed using R statistical software (R 2010 – Version 2.13.1; Suppl. materials 1, 4). We defined color hue labels for the principle colors of interest in this study (i.e. red, magenta, purple, blue, cyan, green, yellow, and orange) as follows. Pure blue (RGB: 0,0,255), red (RGB: 255,0,0) and green (RGB: 0,255,0) are defined by the absence of other RGB values and 255 for the respective color. Magenta (RGB: 255,0,255), cyan (RGB: 0,255,255), and yellow (RGB: 255,255,0) follow an extended PATO definition (Suppl. material 6). Orange is an intermediate value between yellow and red, based on the PATO definition of orange as an intermediate color (RGB: 255,165,0). Purple (RGB: 128,0,128) is an intermediate color whose hue, after conversion to HSV, is equivalent to magenta (300°) at varying levels of saturation or addition of other hues. Since our concern in this publication is the principle hue value, purple and magenta are considered synonymous.

**Figure 4.**
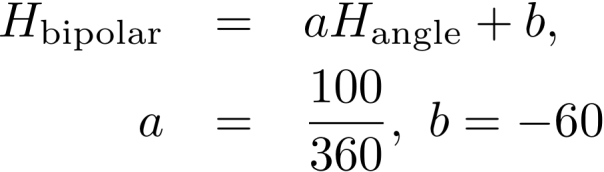
Equation for converting hue values in units degree to hue values as bipolar percent, where blue is near zero.

Color values were converted from Red, Green, Blue color model (RGB) to Hue, Saturation, and Value color model (HSV). Conversion was accomplished using the rgbtohsv library in the R package *grDevices* version 2.13.1 (R 2010). The HSV cylindrical-coordinate representation of color is flattened for analysis by including only the recovered Hue values. Saturation and Value are excluded as Value (i.e. brightness) may be dependent on the computer monitor brightness (Hung and Tsai 2008) and Saturation (addition of black and white) is outside of the scope of this article, as it would add additional complexity to the analysis. Additionally, in order to render results in a linear, rather than 360 degree (i.e. color wheel) representation, and since the majority of the Hue values from the data cluster around blue, all Hue degree values were mapped onto a bipolar linear range of values from -60 to 40, centered near true blue, creating a bipolar range of hues. The equation for converting H_degree_ to H_bipolar_ is represented in Fig. 4.

The basic color hue representations discussed above are outlined in Table 1.

**Figure 5.**
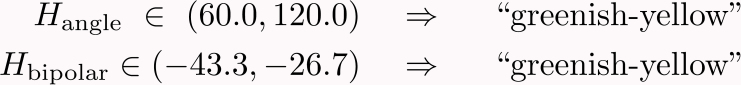
Example of both Hdegree and Hbipolar for proposed hue ranges.

Phenotypic Quality Ontology Uniform Resource Identifiers (URI) are mapped to each hue value. The modifications to existing PATO definitions necessary to fully describe image hues were formally proposed for inclusion in the PATO ontology via the Open Biomedical Ontology SourceForge request account (https://sourceforge.net/p/obo/phenotypic-quality-pato-requests). Additionally, terminology was proposed to represent intermediate color value ranges, and Encyclopedia of Life URI are assigned to these terms at the present time. Each proposed color term corresponds to a range of values between primary hues as in the example of Fig. 5.

The range, or intermediate values, between primary Hues (i.e. red, magenta, purple, blue, cyan, green, yellow, and orange) can be represented in H_degree_, H_bipolar_ or using natural language terms. The representations for the hue range values are outlined in Table 2.

## Results

**Figure 6.**
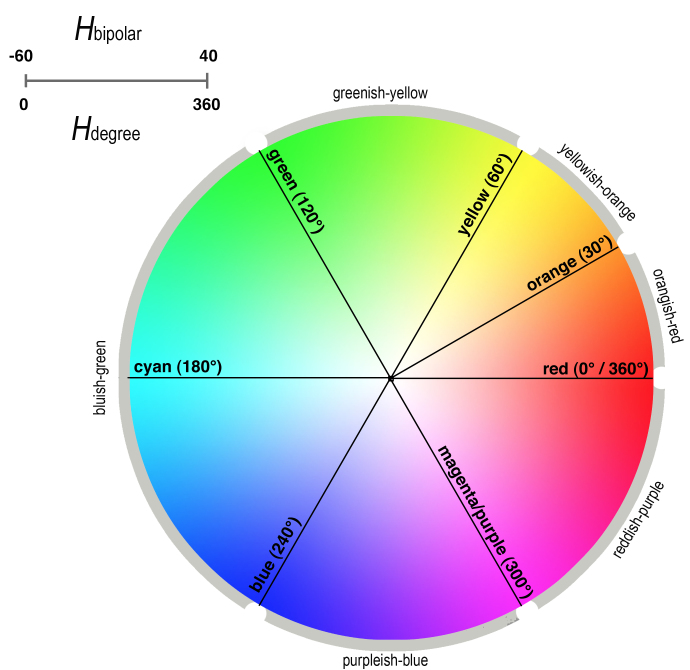
Graphical representation of color Hue values on the color wheel, with modified PATO definitions.

Searching for "blue flowers" from the EOL search interface resulted in 1165 images representing unique specimens, and 182 unique species. 33 species recovered have a common name that contains the word "blue". The reminder of the 149 species were retrieved because the word "blue" occurred somewhere in the description. Based on the analysis described above, records returned using the color name "blue" presented a spectrum of flower colors spanning all angles of the color wheel (Fig. 6).

**Figure 7.**
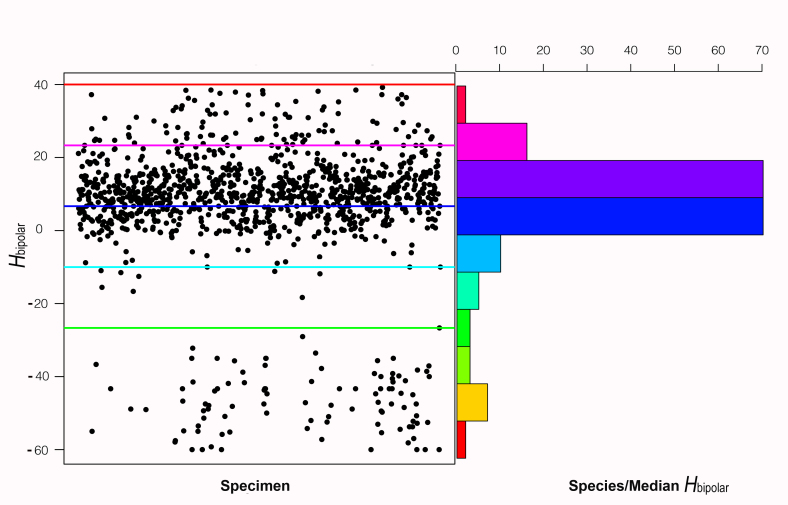
Distribution of hue values returned from "blue flowers" during EOL searching.

Fig. 7 graphically represents the results on the bipolar linear scale. Each point on the scatterplot indicates the hue value H_bipolar_ of one of the sampled images, with the Y-axis being the hue value and the X-axis the unique image number. The colored lines running horizontally across the scatterplot represent the position of pure hues of green, cyan, blue, magenta, and red drawn at their respective values. To the right, the histogram shows the number of species whose median hue value falls within each bin. The color of each bar on the histogram represents the midpoint hue value of the range of hues from each respective bin. The scatterplot in Figure 7 illustrates that the majority of images retrieved by searching for "blue" have hue values that fall between blue and magenta. The majority of the species have a median hue value between 0 and 20, again indicating that most of the flowers from the search results for "blue" are actually blue to magenta in hue value. The median converted HSV value for all images is 251°, corresponding to a H_bipolar_ value of 9.68, and a transitional hue value between blue and magenta (Fig. 6). The outlier hue values below pure green (-26.67) on the graph result from either a white flower described in the search results as "blue" or a flower that includes complementary colors that when averaged resulted in gray.

## Discussion

The Encyclopedia of Life (EOL) collects images from individual users and from online "Content Partners". These Content Partners are varied, ranging from museums and government divisions such as US Fish and Wildlife Service (http://www.fws.gov), to open-access, community driven websites like Flickr (http://www.flickr.com). We surmised from this investigation that, at the present time, researching phenotype data utilizing the present EOL interface is difficult. This is not an observation unknown to EOL, as demonstrated by the recent inclusion of the TraitBank software (Parr et al. 2014a) as a novel means of including controlled vocabularies in searching. For this specific study, each species of flower that was returned in a search result had to be evaluated independently for color. We found that many species were included in the EOL search results because the words "blue" and "flower" both appeared somewhere within the free text descriptive blocks, and did not always represent an accurate account of the flower phenotype as appearing in the associated images. The search function on EOL does not work by searching assigned keywords or tags on images; rather, it searches the entire descriptive content, although filters can be applied to show only search results for taxa, articles, or image descriptions. However, if sorting by image when searching for "blue flower," images are only found if the associated species has the words "blue" and "flower" somewhere in the file name or image description; since not all images have a description or even a descriptive filename, only 218 images result from a filtered search. We suggest further development of controlled vocabulary tagging, utilizing Phenotypic Quality Ontology (PATO; Gkoutos et al. 2005), to augment image descriptions as a method of informing content descriptions. Tags directly associated with images would produce the added benefit of creating specimen level content for EOL.

The ability to control and standardize imaging techniques in as large a resource as EOL is challenging, and this study expected to find variation in flower color within a single species, based on image quality or intraspecific variation. We suggest that the study of color based on EOL images would benefit from the ability to easily access original images that contain color profile information embedded within the image metadata. Color profile information, found in the Exchangeable image file format (Exif), may improve the accuracy of color analysis, as it contains information about the image and camera settings when the photograph was taken (Ricker 2004). Additionally, access to original images reduces the possibility of modifications and enhancements that photographers add during post-processing, which may additionally bias the results. EOL does provide links to the provider of the image, where the original image may be stored. However, there is no way to know the quality of the image from the provider without leaving the EOL website.

A final suggestion for stimulating scientific research utilizing EOL is to encourage data providers to include geo-coordinates for images, as each image is a representation of an individual occurrence of a species. These could be provided either manually or through Exif metadata. For this study, USDA PLANTS was used to acquire the native range of species whose native ranges could not be found in the taxon information available on EOL. However, general species ranges are often vague and typically do not include enough detail concerning specific environmental conditions of the specimen for ecological inference.

A new feature on EOL, released near the end of this project, is the TraitBank. TraitBank is a "searchable comprehensive, open digital repository for organism traits, measurements, interactions and other facts for all taxa across the tree of life" (http://eol.org/traitbank). This novel search tool offers selected search criteria for a number of phenotype attributes. Flower color is one of the searchable traits, with "blue" on EOL being defined sensu PATO. The color annotation originates from the USDA PLANTS database characteristics list, which compiles data "from the scientific literature, gray literature, agency documents, and the knowledge of plant specialists" (http://www.fws.gov, 2013). At the present time, only 8 color options exist on USDA PLANTS (blue, brown, green, orange, purple, red, white and yellow). USDA PLANTS do not define these colors, while EOL appends the PATO definitions. These definitions, while helpful, are still limited. Photoshop displays RGB values using a scale of 0-255. 8-bit images (such as a .jpg file) have 256 possible brightness values, and Photoshop continues to use this standard today. The PATO ontology bases its definitions on color wavelength. Unfortunately, there is no easy or accurate conversion between colors as defined by PATO and color in a Hue, Saturation and Value (HSV) representation as there is no unique mapping between wavelength and RGB. Additionally, some RGB values may be a representation of multiple wavelengths (Berns et al. 2000). Furthermore, only 72 species result from the search for blue flower color using TraitBank, where our search conducted using the general search and narrowing to flower taxa (in North America) resulted in 151 species, which have been called "blue" by at least one source. The number of TraitBank annotated species is lower than our search result at the present time; however, this will likely improve as annotated data is ingested from varied content providers.

Data from this study indicates that the majority of the images sampled have hue values that fall above blue towards magenta, into what might be called "purple" hue rather than "blue" hue. The ability to tag images with a structured vocabulary that includes suggestions of intermediate ranges like "purplish" or "blueish-purple" to differentiate among the colors that fall between magenta and blue rather than magenta and red would be helpful in clarifying color descriptions. We suggest the adoption of a Color Naming System (CNS) terminology with HSV or RGB intermediate values. Assigning natural language terms to discrete ranges of hue values could be used to convert the results from an automatic color picker tool to terms more easily understood by image describers. Further support for the adoption of an expanded color naming system exists in the literature. Berk et al. (1982) demonstrated users annotating colors using RGB, Hue, Saturation and Lightness (HSL) and the Color Naming System (CNS), based on the color lexicon used by the Inter-Society Color Council (ISCC – Berk et al. 1982). The users of the CNS system were significantly more accurate in specifying color than users identifying color with the RGB and HSL numerical systems. The ISCC lexicon is in turn based on the Munsell system (Simon 1997) and the HSL system, another common cylindrical-coordinate representation of points in an RGB color model similar to HSV.

As a further result of this investigation, the image hue value annotations, in the form of a EOL or PATO URI, were returned to EOL TraitBank based on our analysis. Stable URIs associated with ontology classes make it plausible to share phenotype information captured in publication intelligently through the Web (Seltmann et al. 2012). However, to successfully capture the scope of color annotation that is possible, we proposed extending the PATO definitions of color terms to include hue values and color terms representing intermediate ranges of color. The proposed modifications to PATO definitions of color are necessary to describe image hue and were formally proposed for inclusion in the PATO ontology via the Open Biomedical Ontology SourceForge request account (https://sourceforge.net/p/obo/phenotypic-quality-pato-requests). In this request colors not commonly occurring in common language, such as magenta and cyan, are not included; however, these would fit in a future update following the same model we propose. In the interim, EOL has provided stable identifiers for inclusion in our dataset.

### Conclusion

In conclusion, this study revealed that searching for "blue" flowers on EOL returned images that when analyzed represented color values that range from blue to magenta. The PATO terms presently available for use by EOL include violet, blue, cyan, green, purple, light blue, dark blue, and saturated blue in the range of blue descriptors. Studies have shown that humans may be able to best represent color in textual descriptions that include more nuanced variation (i.e. greenish-blue, bluish-purple) than is currently available through PATO. We propose to extend PATO (Suppl. material 6), and available color hue descriptors, to include relative values for blue and other colors. A greater palette for color annotation would aid image description, and supply a richer ontology for color for tagging and assigning phenotype traits, which would make color research using EOL’s resources more refined for scientific research. These tags and traits should also be applied to images directly to aid phenotype research, as species can display a range of traits, and many traits are not yet summarized to their extent in the scientific literature.

## Supplementary Material

Supplementary material 1 Final scripts and graph methods Data type: R Analysis
Brief description: Final scripts and graph methods for figures represented in this study.
File: oo_7570.R Katja C. Seltmann

Supplementary material 2 Image information and data Data type: Excel
Brief description: Master Excel file with metadata about the image. Also includes metadata about the specific imaged specimen, or generalized species metadata when specimen data is not available.
File: oo_8663.xlsx Chantal-Marie Wright, Katja C. Seltmann

Supplementary material 3 Data field definitions Data type: text
Brief description: Data field definitions for the master Excel file (Document 2).
File: oo_8664.docx Chantal-Marie Wright

Supplementary material 4 Tab delimited text file Data type: Image analysis
Brief description: Tab delimited text file used in R scripts.
File: oo_6696.tsv Katja C. Seltmann

Supplementary material 5 RGB values recovered from point analysis of images Data type: RGB values
Brief description: RGB values recovered from point analysis of images. Used in R scripts to convert to HSV.
File: oo_8666.tsv Katja C. Seltmann

Supplementary material 6 Color Terms and URI Data type: URI
Brief description: URIs and definitions of hues as defined by PATO ontology or defined by authors. These terms were proposed for inclusion in PATO, or edits to existing definitions in PATO. Where PATO URIs were not available eol identifiers were included.
File: oo_8667.xls Katja C. Seltmann and Chantal-Marie Wright

## Figures and Tables

**Table 1.** Values for the principle colors of interest as represented in RGB, HSV, H_bipolar_ color models and PATO URI.

Color label	RGB	HSV (H_degree_, S%, V%)	H_bipolar_	PATO Identifier
red	255,0,0	0°,100%,100%	40/-60	http://purl.obolibrary.org/obo/PATO_0000322
magenta	255,0,255	300°,100%,100%	20	http://purl.obolibrary.org/obo/PATO_0000321
purple	128,0,128	300°,100%,50%	20	http://purl.obolibrary.org/obo/PATO_0000951
blue	0,0,255	240°,100%,100%	7	http://purl.obolibrary.org/obo/PATO_0000318
cyan	0,255,255	180°100%,100%	-10	http://purl.obolibrary.org/obo/PATO_0000319
green	0,255,0	120°,100%,100%	-30	http://purl.obolibrary.org/obo/PATO_0000320
yellow	255,255,0	60°,100%,100%	-43	http://purl.obolibrary.org/obo/PATO_0000324
orange	255,128,0	30°,100%,100%	-52	http://purl.obolibrary.org/obo/PATO_0000953

**Table 2.** Range hue values for proposed intermediate colors as represented in RGB, HSV, Hbipolar color models, and PATO or EOL URI.

Color	H_degree_ Range	H_bipolar_ Range	EOL or PATO Identifier
greenish-yellow	60°,120°	-43,-27	http://purl.obolibrary.org/obo/PATO_0001941
yellowish-orange	30°,60°	-51,-43	http://purl.obolibrary.org/obo/PATO_0001944
orangish-red	0°,30°	-60(0),-51	http://eol.org/schema/terms/orangish-red
reddish-purple	300°,360°	23,-60(0)	http://eol.org/schema/terms/reddish-purple
purplish-blue	240°,300°	7,23	http://eol.org/schema/terms/purplish-blue
bluish-green	120°,240°	-27,7	http://eol.org/schema/terms/bluish-green
