# Supplementary material for: Usage patterns of blue flower color representation by Encyclopedia of Life content providers
Source: Biodivers Data J. 2014 Aug 11;(2):e1143. doi: 10.3897/BDJ.2.e1143 (PMC4152829; doi:10.3897/BDJ.2.e1143)
Supplement: Supplementary material 3 — Data field definitions [file biodiversity_data_journal-2-e1143-s003.docx]

**Column Definitions for "master analysis color" spreadsheet**

**Species** – Genus and species name from EOL

**Family** – Taxonomic family from EOL

**Common_Name** – Common non-taxonomic name from EOL

**Lat** – Specimen latitude, from EOL entry or Google Earth

**Long** – Specimen longitude, from EOL entry or Google Earth

**Elevation_meters** – Specimen elevation, from EOL entry or Google Earth

**Alpine** – Alpine/high altitude locality or not

**Locality** – Locality information for specimen, from EOL

**Locality_Notes** – Pertinent locality information

**R** – Red (RGB)

**G** – Green (RGB)

**B** – Blue (RGB)

**Link** – Link to specimen entry on EOL

**Notes** – Pertinent information about specimen entry

**H** – Hue (HSV)

**S** – Saturation (HSV)

**V** – Value (HSV)

**h_degree -** Hue value converted to degrees

**s_percent -** Saturation value represented as percent/100

**v_percnet -** Value represented as percent/100

**converted_hue -** Transformed hue value

**specimen_number -** Unique counting number for each specimen examined.

**Blue in Name?** – Term “blue” in the common name? yes or no

**Purple in Name?** - Term “purple” in the common name? yes or no

**URI** - Uniform resource identifier for color term

**Locality Terms**

**Introduced** - growing wild but not in a native habitat

**Non-native** - not native to the area where photographed; unsure if specimen pictured is wild or cultivate

**Cultivated** - in a greenhouse, garden, or other captive setting; used for species blueness analysis but not direct altitude correlation
